# Benefits of SGLT2 inhibitors in arrhythmias

**DOI:** 10.3389/fcvm.2022.1011429

**Published:** 2022-10-20

**Authors:** Jinghan Gao, Genlong Xue, Ge Zhan, Xinying Wang, Jiatian Li, Xiaolei Yang, Yunlong Xia

**Affiliations:** Department of Cardiology, The First Affiliated Hospital of Dalian Medical University, Dalian, Liaoning, China

**Keywords:** SGLT2 inhibitors, arrhythmia, electrical remodeling, structural remodeling, mechanisms

## Abstract

Some studies have shown that sodium-glucose cotransporter (SGLT) 2 inhibitors can definitively attenuate the occurrence of cardiovascular diseases such as heart failure (HF), dilated cardiomyopathy (DCM), and myocardial infarction. With the development of research, SGLT2 inhibitors can also reduce the risk of arrhythmias. So in this review, how SGLT2 inhibitors play a role in reducing the risk of arrhythmia from the perspective of electrical remodeling and structural remodeling are explored and then the possible mechanisms are discussed. Specifically, we focus on the role of SGLT2 inhibitors in Na^+^ and Ca^2 +^ homeostasis and the transients of Na^+^ and Ca^2 +^, which could affect electrical remodeling and then lead to arrythmia. We also discuss the protective role of SGLT2 inhibitors in structural remodeling from the perspective of fibrosis, inflammation, oxidative stress, and apoptosis. Ultimately, it is clear that SGLT2 inhibitors have significant benefits on cardiovascular diseases such as HF, myocardial hypertrophy and myocardial infarction. It can be expected that SGLT2 inhibitors can reduce the risk of arrhythmia.

## Introduction

With the notion that they lower blood glucose levels, chemists were able to successfully isolate several sodium-glucose cotransporter (SGLT) inhibitors in the 19th century (1). It was understood that, independent of insulin, SGLT inhibitors modify specific phases of kidney metabolism to promote glycosuria, consequently lowering blood glucose (2). SGLT proteins exist in two forms, SGLT1 and SGLT2. Further research revealed that SGLT2 proteins are found mostly in the S1 segment of the proximal convoluted tubule (PCT) and account for approximately 90% of glucose reabsorption (3). SGLT1 proteins are located in the S2/S3 segment of PCT and facilitate reabsorption of the remaining glucose (4). Therefore, blocking SGLT proteins to reduce glucose reabsorption may be a novel therapeutic strategy. Being among the newest hypoglycemics on the market, SGLT2 inhibitors have been extensively studied and demonstrated to significantly reduce the likelihood of suffering cardiovascular events (5). Empagliflozin (EMPA), for example, is a highly specific SGLT2 inhibitor, that has been shown to significantly reduce cardiovascular mortality (6). The EMPA-REG OUTCOME^®^ trial (ClinicalTrials.gov number, NCT01131676), the first trial to report the effects of empagliflozin on cardiovascular outcomes, indicated that empagliflozin can significantly reduce cardiovascular and all-cause mortality by 38 and 32%, respectively (7). Scientific investigation has shown that other SGLT2 inhibitors, such as canagliflozin (1) and dapagliflozin, also have protective effects on the cardiovascular health of diabetes mellitus (DM) type 2 sufferers (2). Canagliflozin can significantly reduce the incidence rate, which is similar to the composite of death from cardiovascular causes, non-fatal myocardial infarction or non-fatal stroke (1). A few years after the EMPA-REG OUTCOME trial, the DECLARE–TIMI 58 trial showed that dapagliflozin has a beneficial effect on non-diabetic patients with heart failure (HF) (3). It has been established that SGLT2 inhibitors reduce the incidence of cardiovascular events by preventing HF (4, 5). In addition, it has also been shown that inhibition of SGLT2 can reduce atrial natriuretic peptide (ANP) and B-type natriuretic peptide (BNP) in zebrafish models of heart failure (6). Borghetti et al. showed that SGLT2 inhibitors prevent the deterioration of cardiac function in various models of HF (7). Further research revealed that SGLT2 inhibitors could reduce the risk of cardiovascular disease in both diabeticsand non-diabetic individuals (3). The Dapagliflozin and Prevention of Adverse Outcomes in Heart Failure Trial (DAPA-HF trial) (ClinicalTrials.gov number, NCT03036124) demonstrated that treatment with dapagliflozin definitively reduced the risk of precipitated HF or death from cardiovascular causes in patients with HF. And with the development of clinical research, a risk association for AF was observed in the study of Shao et al. between dapagliflozin and empagliflozin (8).

In experimental research, empagliflozin showed a protective effect on diastolic function and cardiac hypertrophy in mice (9). In a rat model with DM, empagliflozin attenuated cardiac inflammation by activating AMPK signaling pathway and promoting autophagy in cardiomyocytes (10). On the other hand, dapagliflozin reduced inflammation by attenuation of NOD-like receptor 3 (NLRP3), interleukin 1β (IL1β), and interleukin-6 (IL6) through the activation of AMP-activated protein kinase (AMPK) pathways (11). Furthermore, dapagliflozin has demonstrated the ability to activate the mTOR pathway, slowing the progression of dilated cardiomyopathy (DCM) as a result (12). Empagliflozin ameliorated LV diastolic function in a DCM model by suppressing calcium/calmodulin-dependent protein kinase II (CaMKII) Thr286, a protein kinase which can retard the phosphorylation of ryanodine receptors (13). In a diabetic mouse model, empagliflozin stimulated sarcoplasmic ATPase2a (SERCA2a) function thus enabling the Ca^2 +^ homeostasis required to improve diastolic function (14). A similar study revealed that empagliflozin can reduce the degree of myocardial fibrosis in diabetic mice by attenuating the transforming growth factor-Smad pathway (15). Yumei Ye et al. demonstrated that in cardiac fibroblasts, dapagliflozin down regulated the NLRP3 inflammasome (16).

From certain reports and studies, SGLT2 inhibitors appear to have an antiarrhythmic effect. Here, how SGLT2 inhibitors plays a protective effect in arrhythmia is discussed.

## SGLT2 inhibitors in arrhythmia

As one of the most prevalent chronic diseases in the world, diabetes mellitus (DM) has been extensively investigated, from basic questions like prevention and pathogenesis to the challenging issues of pharmacological management and lifestyle modification (17). DM is associated with various complications including cardiovascular, cerebrovascular and renal disease (18). Therefore, it is imperative that improved cost-effective preventive and treatment solutions are continuously developed to combat the disease. The glucose-lowering function of SGLT2 inhibitors is a result of reduced glucose reabsorption due to SGLT2 cotransporter inhibition in the kidney (19). It has been established in past literature that T2DM is closely related to atrial fibrillation (AF) (20). Recently, some studies have revealed that SGLT2 inhibitors can significantly reduce the incidence rate of AF (21). The physiological mechanisms by which this reduction is achieved are related to myocardial remodeling arising from inflammation-induced cardiac fibrosis (22). Changes in atrial electrical remodeling have also been linked with reduced arrhythmogenesis (23).

Studies spanning decades have established that, regardless of the form and degree of electrical or structural remodeling in the ventricles, dysrhythmias can develop (24). The development and frequency of arrhythmias is closely related to various electrophysiological abnormalities, including ectopic automaticity, triggered by early after-depolarizations, and reentry in cardiac tissue (25). Ectopic beats of the atrium or ventricle usually stem from their respective ectopic automaticity. On the other hand, after depolarizations are usually triggered by an imbalance in Ca^2 +^ homeostasis, resulting in early or delayed after-depolarizations (26). Reentrant arrhythmias have distinct mechanisms usually involving reentry circuits, an anatomical block and a slowed conduction pathway (27).

However, the risk of arrhythmias is generally inflated by fibrosis-induced cardiac remodeling, hypertrophy and dysfunction (28). As previously mentioned, electrical and morphological remodeling are two key forms of remodeling that contribute to arrhythmogenesis. This study reviews both forms and examines how SGLT2 inhibitors reverse them.

### Clinical evidence

In past clinical studies, SGLT2 inhibitors have demonstrated outstanding cardiovascular benefits in patients with DMT2, particularly those with comorbid HF (4). In EMPA-REG OUTCOME study (29), empagliflozin reduced the incidence of HF by a staggering 35% (4). The CANVAS study (ClinicalTrials.gov, numbers, NCT01032629 and NCT01989754) confirmed that SGLT2 inhibitors could indeed significantly reduce the incidence of HF (hazard ratio: 0.67; 95% confidential interval: 0.52–0.87) (1). Dapagliflozin, one of the older SGLT2 inhibitors, has been shown to significantly reduce the morbidity and mortality of cardiovascular disease in HF patients with or without DM (hazard ratio: 0.75 and 0.73 respectively) (ClinicalTrials.gov numbers: NCT03036124) (30), improve biomarkers in HF and better patients’ quality of life (ClinicalTrials.gov numbers: NCT03036124) (31). For these reasons there is a consensus that SGLT2 inhibitors preserve cardiovascular function, keeping heart failure at bay. A recent study showed that empagliflozin could also reduce the blood pressure in DMT2 patients (32). Therefore, it is plausible that SGLT2 inhibitors slow the development of HF by lowering blood pressure to improve the load of heart. In these cases, improved diastolic function was observed, attributable to reduced preload. Similar studies revealed that only hemodynamic changes, and not physiological changes, contribute to the drug-induced improvement in diastolic function (33).

It has been established that patients with HF tend to develop atrial fibrillation (AF) (34), so it is clear that preventing HF would also retard the development of AF. Therefore, some studies suggested that SGLT2 inhibitors did not directly have protective effects on arrhythmias (35). SGLT2 inhibitors have indirect protective effects on arrhythmias, such as reducing the risk of AF suffering a myocardial function (36). A study suggested that SGLT2 inhibitors may reduce the risk of AF (35). Meanwhile, Zelniker et al. showed that compared with the group without dapagliflozin, the risk of atrial fibrillation in the group receiving dapagliflozin was reduced by 19%, and the total number of atrial fibrillation events was significantly reduced (37). According to the study from Li et al., SGLT2 inhibitors can significantly reduce the likelihood of serious adverse events (SAEs) in AF (risk ratio: 0.83; 95% confidence interval 0.71–0.96) (38). In addition, these agents were associated with a lower risk of new-onset AF in comparison to other hypoglycemic agents (hazard ratio: 0.61; 95% confidential interval: 0.50–0.73) (39). Dapagliflozin can also significantly reduce the risk of supraventricular and atrial tachycardia (37). On a positive note, the antiarrhythmic effect of dapagliflozin was independent of age, sex, BMI, HbA1c, and eGFR (37). Owing to the hypotensive abilities of SGLT2 inhibitors, it is conceivable that they reduce the risk of AF considerably (40). A meta-analysis published recently suggested that SGLT2 inhibitors specifically reduced the risk of ventricular tachycardia (VT). In the analysis report, only the group receiving empagliflozin was deemed to be at a lower risk of suffering VT. Therefore, SGLT2 inhibitors may in fact may notably protect these patients from VT (41). However, evidence from prospective trials showing how SGLT2 inhibitors primarily deter arrhythmia was lacking. Nonetheless, it can be safely stated that SGLT2 inhibitors have specific antiarrhythmic properties.

Obesity appears to be a another key risk factor for AF, a hypothesis backed by the Framingham Heart Study (42). Clinical studies have confirmed that SGLT2 inhibitors can reduce the risk of AF in patients with T2DM (40) which is associated with the loss of weight (43). Meanwhile, the Framingham study, a long term cohort study, has reported that the deposition of pericardial adipose tissue is associated with the arrhythmogenesis of atrial fibrillation (odds ratio: 1.28; 95% confidential interval: 1.03–1.58) (44). Notably, dapagliflozin and empagliflozin reduce body fat (45) and epicardial fat thickness (46, 47), consequently impeding arrhythmogenesis in diabetics. Accordingly, SGLT2 inhibitor-mediated reduction in epicardial fat volume staves off AF, although the exact mechanism thereof remains unknown. Table 1 summarizes related studies regarding the SGLT2 inhibitors and risk of arrhythmias.

**TABLE 1:** The impact of SGLT2 inhibitors on the risk of arrhythmias.

References	Study design	Research population	Main finding
Curtain et al. (48)	Cross-sectional	Patients with heart failure	Dapagliflozin reduced the risk of any serious ventricular arrhythmia in patients with heart failure (HR 0.79)
Li et al. (41)	Meta-analysis	General population	SGLT2 inhibitor was associated with a lower risk of AF (RR 0.82, 95% CI 0.70–0.96) and VT (RR 0.73, 95% CI 0.53–0.99)
Zhou et al. (35)	Meta-analysis	General population	Canagliflozin has no direct effects on the incidence of AF (HR 0.76 [95% CI, 0.53–1.10]; *P* = 0.15)
Zhou et al. (49)	Cohort	T2DM patients	Canagliflozin did not significantly reduce the risk of AF (HR 0.84 [95% CI, 0.64–1.12]; *P* = 0.23)
Zelniker et al. (37)	Cross-sectional	T2DM patients	Dapagliflozin significantly reduce the risk of AF/AFL events [HR 0.81(95% CI, 0.68–0.95); *P* = 0.009) regardless of the patient’s previous history of AF
Kovesdy et al. (50)	Cohort	T2DM patients	Atrial fibrillation [HR 2.97 (CI 1.51, 5.84)] is related to adverse events of SGLT2 inhibitors in patients with diabetic kidney disease
Chen et al. (51)	Cohort	T2DM patients	SGLT2 inhibitors could significantly lower the risk of new-onset arrhythmias (aHR 0.830; 95% CI 0.751–0.916; *P* = 0.0002)
Bohm et al. (52)	Cross-sectional	T2DM patients	EMPA can reduce the risk of HF in patients with AF
Bonora et al. (53)	Cross-sectional	T2DM patients	Compared with other drugs, SGLT2 inhibitors can reduce the occurrence of AF more effectively
Wang et al. (54)	Meta-analysis	T2DM patients with HF	SGLT2 inhibitors can significantly reduce the incidence of AF (OR 0.8) and reduce the incidence of arrhythmia (OR 0.86)
Kwon et al. (55)	Cohort	T2DM patients with AF	SGLT2 inhibitors can significantly reduce the incidence of hospitalization for heart failure (HR 0.70; 95% CI 0.53 to 0.93; *P* = 0.012)

T2DM, Type 2 diabetes; AF, atrial fibrillation; HF, heart failure; OR, odds ratio; HR, hazard ratio; CI, confidence interval.

### Effects of SGLT2 inhibitors on ion homeostasis

**FIGURE 1:**
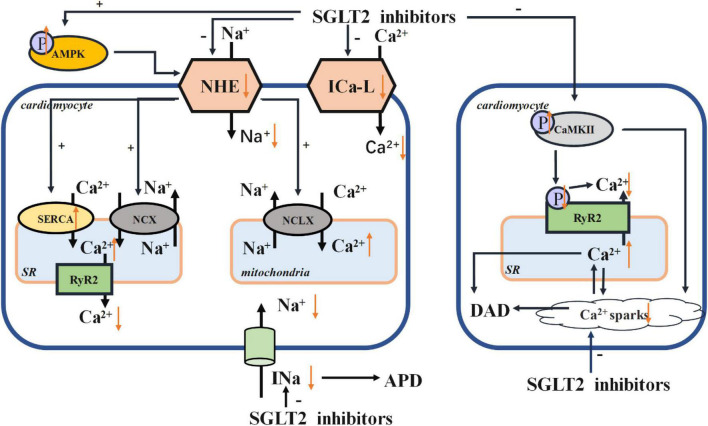
Electrical remodeling. AMPK, adenosine monophosphate kinase; NHE, Na^+^ /H^+^ exchanger; NCX, Na^+^ /Ca^2 +^ exchanger; NCLX, Na^+^ /Ca^2 +^ exchanger; SR, sarcoplasmic reticulum; SERCA, sarco/endoplasmic reticulum Ca^2 +^ -ATPase; RyR, ryanodine receptor; ICa-L, L-type calcium channel; CaMKII, calcium/calmodulin-dependent protein kinase II; APD, action potential duration; DAD, delayed after depolarization.

Na^+^ and Ca^2 +^ homeostasis are the key players in the cardiac cycle. The intracellular concentrations of Na^+^ and Ca^2 +^ are influenced by the Na^+^ /H^+^ exchanger (NHE), Na^+^ /Ca^2 +^ exchanger (NCX), Na^+^ /Ca^2 +^ exchanger (NCLX), sarco/endoplasmic reticulum Ca^2 +^ -ATPase (SERCA), and L-type calcium channel (ICa-L). SGLT2 inhibitors may directly affect the function of NHE, which could impact NCX and NCLX (56). SGLT2 inhibitors can enhance the phosphorylation of AMPK, thus suppressing the activity of NHE. SGLT2 inhibitors can increase the expression of SERCA2a, which improves contractility (57), subsequently decreasing the concentration of Ca^2 +^ in cardiomyocytes. SGLT2 inhibitors can also improve the activity of phospholamban (PLN), resulting in the phosphorylation of calcium/calmodulin-dependent protein kinase II (CaMKII) and further affects sarcoplasmic release of Ca^2 +^. In this way, SGLT2 inhibitors prevent calcium overload and thus reducing the likelihood of arrhythmias. In this review, we have summarized several mechanisms of SGLT2 inhibitors acting on ion homeostasis and electrical remodeling in Figure 1.

#### Effects of SGLT2 inhibitors on sodium transient

Early after-depolarizations (EADs) and delayed after-depolarizations (DADs) are likely triggers of dysrhythmias. A decrease in repolarizing currents such as rectifier outward K^+^ currents or an increase in depolarizing currents such as late sodium current (I_*Na*_) would trigger prolonged action potential duration (APD), which in turn induces EADs (58). In previous studies, the increase of late I_*Na*_ and Na^+^ influx solely accounted for upregulation of NHE activity (59). Moreover, by activating the activity of NHE1, the cytosolic concentrations of Na^+^ and Ca^2 +^ were increased. As a result, the concentration of Ca^2 +^ in mitochondria was decreased and eventually induced the cardiomyocyte apoptosis and the instigation of HF (60). Conversely, the NHE1 inhibitor cariporide has been shown to protect cardiomyocytes (61). Jiang et al. reported that empagliflozin could reduce the cytoplasmic concentrations of Na^+^ and Ca^2 +^, and also reverse the harmful effects of NHE1 overexpression in cardiomyocytes (60). This finding suggested that empagliflozin can protect myocardial function *via* a cardiac-specific NHE1. Both dapagliflozin and canagliflozin can also preserve cardiac function by reducing the concentration of cytosolic Na^+^ ([Na^+^]c) and the activity of cardiomyocyte specific NHE1 (62). Collectively, these studies point to SGLT2 inhibitors providing myocardial protective effects *via* regulation of intracellular Na^+^ ([Na^+^]i). In particular, a decrease in [Na^+^]i could prevent progression from ventricular tachycardia to life-threatening ventricular fibrillation. This phenomenon supports the claim increased Na^+^ concentration facilitates arrhythmogenesis of ventricular arrhythmias (63). Concurrently, At the same time, raised [Na^+^]i levels will eventually incite generation of reactive oxygen species (ROS) by mitochondria (64), compromising cardiac function as a result. Activation of late Na^+^ current will further exacerbate intracellular Na^+^ overload and disrupt contractile and electrical activity all the more. Some studies have suggested that empagliflozin can significantly attenuate late I_*Na*_ without affecting peak I_*Na*_, thus imparting more protection to the heart (65). There is strong evidence that the induction of late I_*Na*_ is closely related to arrhythmias (61) and late I_*Na*_ could further fluctuate APD, which dictates the likelihood of arrhythmias (66). Worth noting, is that the dispersion of APD, the triggering of arrhythmias, and the induction of ventricular tachycardia can be attenuated by impeding the generation of late I_*Na*_ (67). SGLT2 inhibitors are potent late I_*Na*_ inhibitors and are highly selective for late I_*Na*_ (61). This signifies that SGLT2 inhibitors could markedly decrease the risk of arrhythmias.

#### Effects of SGLT2 inhibitors on calcium transients

It is clear that Ca^2 +^ and Na^+^ homeostasis are the key point for heart rhythm and cardiac signal pathways (68, 69). Cardiomyocytes tightly link Ca^2 +^ handling to Na^+^ handling by activating NCX and mitochondrial NCLX (70). Cardiac NCX, the central efflux pathway of Ca^2 +^ from the mitochondria, extrudes intracellular Ca^2 +^ into the extracellular space, ensuring levels of intracellular Ca^2 +^ are regulated (71). Several studies have shown that inhibition of NCLX activity raises the concentration of intracellular Ca^2 +^, thereby preventing NAD(P)H oxidation and reducing mitochondrial ROS emissions (72). On this basis, NCLX can curb myocardial hypertrophy and preserve the normal contractile function, thereby reducing the occurrence of ventricular arrhythmias (72). NHEs and NCX often interact, meaning that Na^+^ concentration can have a direct impact on the Ca^2 +^ concentration (10, 73).

In recent research, dapagliflozin decreased intracellular calcium transients, and the expression of voltage-dependent ICa-L, NCX, and NHE was reduced in dapagliflozin treatment (74). Some studies have shown that inhibition of ICa-L function can impede the entry of extracellular Ca^2 +^, resulting in a decreased levels of intracellular Ca^2 +^ (75). It has also been learned that the density of ICa-L in ventricular myocytes of diabetics on other treatments was less than that in those receiving empagliflozin (76). The reversal of the density of ICa-L in cardiomyocytes treated with empagliflozin improved [Ca^2 +^]i transients and sarcoplasmic Ca^2 +^ contents (76). Baartscheer et al. reported that empagliflozin has the ability to inhibit NHE activity, facilitating the reduction sodium and calcium levels within the cardiomyocytes (56). In light of these works, the protective effect of empagliflozin on the myocardium can be directly attributed to consistent ion homeostasis within cardiomyocytes (77). In this investigation, it was reported that empagliflozin prevented intracellular Na^+^ overload and myocardial oxidative stress by reducing [Na^+^]i and restoring mitochondrial Ca^2 +^ handling (77). And the increase of [Ca^2 +^]m also reduced the incidence of ventricular arrhythmias and sudden cardiac death (72). Indeed, intracellular Na^+^ overload can facilitate cytoplasmic Ca^2 +^ handling and activate the reverse mode Na^+^ /Ca^2 +^ exchange function of NCX, thus promoting Ca^2 +^ influx and cytosolic Ca^2 +^ transient amplitude (78). Empagliflozin can revive the NCX and stimulate the reverse-mode function of NCX, playing its myocardial protective role as a result (76). However, empagliflozin can hinder leakage of Ca^2 +^ into the SR, which may lead to Ca^2 +^ deficit (76). Moreover, empagliflozin can also change the AP morphology (76). Empagliflozin can stimulate expression of the SERCA2a protein and then attenuate the decrease in Ca^2 +^ stores with prolonged [Ca^2 +^]i decay (14).

#### Effects of SGLT2 inhibitors on calcium/calmodulin-dependent protein kinase II

Findings from past research show that DADs could induce arrhythmias. And this process is usually initiated by spontaneous release of Ca^2 +^ from ryanodine receptors (RyR)2, resulting in calcium overload that induces arrhythmias (79). Mustroph et al. revealed that empagliflozin could reduce Ca^2 +^ spark frequency and increase SR Ca^2 +^ load, which are key conditions for setting arrhythmias in motion. It was also affirmed that, at a clinical dose, empagliflozin could reduce CaMKII activity (80). In past research, phosphorylation of PLN, one of the most important CaMKII phospho-sites, was significantly slowed by empagliflozin (5). Because CaMKII can phosphorylate RyR2, further SR Ca^2 +^ release and Ca^2 +^ sparks are also probable (81). Both changes in calcium concentrations generate a depolarizing Na^+^ influx (Iti), which may trigger DAD (82). On the other hand, it is possible that SGLT2 inhibitors indirectly suppress late I_*Na*_ by CaMKII (79). It is also worth noting that treatment with empagliflozin can suppress the Ca^2 +^ sparks arising from transient release of Ca^2 +^ (83).

Increased CaMKII expression and oxidation plays a critical role in the electrophysiological changes that characterize arrhythmogenesis. Therefore, the claim that SGLT2 inhibitors exert antiarrhythmic effects *via* inhibition of CaMKII can be safely made (84, 85). A similar study showed that dapagliflozin attenuated the upregulation of NHE through AMPK activation, validating the claim that SGLT2 inhibitors are similar to NHE inhibitors and have antiarrhythmic effects (86).

### Effects of SGLT2 inhibitors on cardiac structure

**FIGURE 2:**
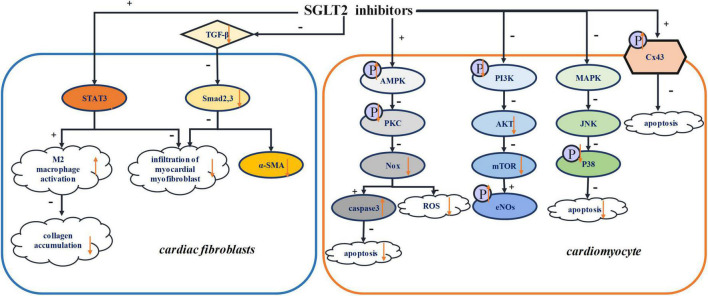
Structural remodeling. STAT3, signal transducer and activator of transcription 3; TGF-β, transforming growth factor-β; Smad2,3, mothers against decapentaplegic homolog 2,3; α-SMA, α-smooth muscle actin; AMPK, adenosine monophosphate kinase; PKC, protein kinase C; Nox, NADPH oxidase; PI3K, phosphatidylinositol-4,5-bisphosphate 3-kinase; Akt, serine/threonine-protein kinase; mTOR, mammalian target of rapamycin; eNOs, endothelial nitric oxide synthase; MAPK, Mitogen-activated protein kinase; JNK, Jun N-terminal kinase; Cx43, connexin 43.

Previous studies have shown that, *via* their diuretic and natriurtic effects, SGLT2 inhibitors consistently reduce preload, thus shielding the body from cardiovascular disease (87, 88). Moreover, remodeling of individual cardiomyocytes leads to the proliferation of fibroblasts and the production of extracellular matrix, culminating in morphological changes within the hearts (89). This manifests in rhythm irregularities such as atrial fibrillation (AF) (90, 91). Other studies report that SGLT2 inhibitors can reverse ventricular remodeling and reduce cardiac afterload by lowering arterial pressure (92, 93). Reduction in oxidative stress has also been attributed to SGLT inhibitors, an effect that impedes myocardial fibrosis, preserving cardiac contractility as a result (94). In this section, the mechanisms by which SGLT2 inhibitors mitigate structural remodeling of the heart are discussed. Figure 2 summarized the various structural remodeling mechanisms associated with SGLT2 inhibitors in inducing arrhythmias, including inflammation, fibrosis, oxidative stress and apoptosis.

#### Effects of SGLT2 inhibitors on cardiac fibrosis and inflammation

SGLT2 inhibitors can lower cardiac workload and improve left ventricular function (95). It has been put forward that SGLT2 inhibitors not only reduce fibrotic scars, but also increase LV wall thickness (60) and prevent AF inducibility in diabetic rats (96). Multiple studies have shown that AF is often closely linked with cardiac fibrosis in multiple animal models, affirming that myocardial fibrosis is indeed an important substrate in the development of AF (89). Further research reveals that myocardial fibrosis can trigger the generation of reentry circuits in myocardium, thus accelerating cardiac automaticity and hinder cation potential conduction, and then promote the occurrence of arrhythmias (97).

Tissue fibrosis and inflammation are instrumental in the progression of cardiac remodeling (98). According to a recent study, dapagliflozin activates M2 macrophage infiltration, inhibiting myocardial collagen synthesis and reducing proliferation of myofibroblasts in the border zone as result, which prevent the depolarization required for arrhythmogenesis. Further studies showed that dapagliflozin increases the activity of signal transducer and activator of transcription 3 (STAT3), which is a key transcription factor for minimizing myocardial myofibroblast infiltration (99). This evidence confirms that STAT3 signaling is critical for regulating myocardial fibroblast infiltration. By activating STAT3, the signal pathway of STAT3 would be attenuated, thereby promoting the activation of M2 macrophage and reducing the infiltration of myocardial myofibroblasts (100). Additionally, dapagliflozin was shown to promote M2 macrophage polarization *via* STAT3 signaling and then protect cardiomyocytes (99).

It has been indicated that empagliflozin markedly slows myocardial fibrosis in an animal model post myocardial infarction (101). Both fibrosis and the expression of fibrosis marker, such as collagen 1 and α-smooth muscle actin, were reduced by empagliflozin treatment. In addition, transforming growth factor-β (TGF-β) and extracellular matrix remodeling, which were significantly attenuated by empagliflozin, can induce the activation of fibroblasts (102). Dapagliflozin can attenuate cardiac remodeling, activation of the NLRP3 inflammasome, fibrotic activity, and LV impairment. However, the specific mechanism by which SGLT2 inhibitors influence NLRP3 inflammation and fibrosis is not clear. However, the anti-inflammatory and antifibrotic effects seem to be unrelated to glucose homeostasis and SGLT2 proteins (16). Ye et al. showed that dapagliflozin significantly increased AMPK levels and its increase could be blocked by AMPK inhibitors. Therefore, it can be concluded that the anti-inflammatory power of SGLT2 inhibitors is dependent on the AMPK pathway (16). This may also suggest that AMPK accounts for the protective effects of SGLT2 inhibitors. Several studies have shown that empagliflozin can promote the phosphorylation of AMPK, which would be less expressed in the model post myocardial injury (103). In addition, empagliflozin increased the AMP/ATP ratio, which would favor the activity of AMPK, ergo delivering its cardiovascular protective effects (104). For these reasons, an investigation of the direct or indirect effects of SGLT2 inhibitors on AMPK signaling pathways is of the essence.

Another experiment has shown that dapagliflozin could prevent cardiac remodeling by inhibiting c-Jun N-terminal kinase (JNK) and P38, two major proteins of mitogen-activated protein kinase (MAPK) pathways (100). It is known that the MAPK pathway plays a critical role the cellular processes that contribute to cardiac remodeling, particularly apoptosis and fibrosis (105). On the other hand, SGLT2 inhibitors can slow cardiac fibrosis by inhibiting NADPH oxidase enzyme family (99), which is related to oxidative stress. Moreover, empagliflozin could regulate the inflammation by reducing myocardial IL-6 and iNOS (106). Empagliflozin can significantly suppress the activity of TNF-α and iNOS (103). And dapagliflozin can inhibit inflammation by reducing the levels of inflammatory cytokines IL-6 and tumor necrosis factor α (TNF-α) and activating the NO-cGMP-PKG pathway (94). In other research, microvascular inflammation, involving the inflammatory mediators ICAM-1, VCAM-1, TNF-α, and IL-6, was higher in samples of myocardial tissue that showed cardiomyopathy with preserved ejection fraction. Empagliflozin significantly attenuated this phenomenon (107).

#### Effects of SGLT2 inhibitors on oxidative stress

Oxidative stress is a critical factor in cardiac remodeling (108). It has been reported that oxidative stress can induce arrhythmias by promoting cardiac fibrosis and constant changes in the electrophysiological properties of the heart (109, 110). It has also been claimed that oxidative stress could increase vulnerability to arrhythmias (111).

SGLT2 inhibitors can counteract oxidative stress and slow myocardial fibrosis (9, 112). In past research, AMPK inhibited ROS by suppressing the phosphorylation of protein kinase C (PKC) (107), thus inhibiting the activation of NADPH oxidase (Nox) (113). Nox is one of the most important sources of ROS (114), and empagliflozin can attenuate oxidative stress in that way. In some studies, dapagliflozin has been reported to mitigate oxidative stress through AMPK/PKC/Nox signaling pathways (115). In other research, with cardioprotective function, Nrf2/HO-1 has antagonistic effect on oxidative stress (116). And Li et al. suggested that empagliflozin could reduce the occurrence of oxidative stress by downregulating the expression of NOX4 and activating Nrf2/ARE signaling pathway (117). Dapagliflozin suppressed zinc transporter 7 (ZnT7), which led to Zn^2 +^ efflux in cardiomyocytes. And it increased ZIP7, which caused Zn^2 +^ to influx into cardiomyocytes (10). The upregulation of Zn^2 +^ in cardiomyocytes had an antioxidative effects on cardiac muscles.

#### Effects of SGLT2 inhibitors on apoptotic pathways

It has been established that cardiomyocytes are terminally differentiated cells, and their massive death can lead to severe structural and functional defects. The death of cardiomyocyte usually occurs by apoptosis and necrosis. With extensive research, novel mechanisms of cell death have been discovered, such as necroptosis, pyroptosis, methuosis, and autophagy (118–120). Autophagy is a natural conservative process that promotes degradation of protein and the recycling of damaged cytoplasmic components in response to various stimuli such as stress, ischemic injury, and infection. However, the mechanisms of autophagy signals and regulation are still unclear (121–123). Similarly, the relationship between autophagy and acute myocardial infarctions is still controversial (124, 125). Both inhibition and activation of autophagy have been shown to reduce the size of myocardial infarction (60) and improve ventricular remodeling after myocardial infarction (126). In fact, autophagy is an essential process for maintaining the normal cardiac function. However, in abnormal conditions, excessive autophagy causes extreme destruction of cellular material and cardiomyocyte death (127). Recently, it has been suggested that empagliflozin may play a protective role by inducing cardiomyocyte autophagy (128, 129). In some research, empagliflozin reduced the detrimental effects of autosis (130). Matsui et al. suggested that, in response to glucose deprivation (GD), pretreatment with SGLT2 inhibitors improved cardiomyocyte survival and induced autophagy by activating the AMPK signaling pathway and inactivating the mechanistic target of rapamycin (mTOR) (130). AMPK-dependent signaling pathway can promote autophagy in ischemic cardiomyocytes (127). The maintenance of a high level of ATP/ADP ratio is essential for maintaining normal cardiomyocyte function (131). Jiang et al. indicated that, by playing a protective role in cardiomyocyte function, empagliflozin could mitigate GD-induced damage to cardiomyocytes. And it can also attenuate the cardiomyocyte apoptosis induced by GD (60). Moreover, dapagliflozin could suppress cardiomyocyte apoptosis by downregulating AMPK and upregulating caspase3 (115). The downregulation of PKC and Nox2 could activate caspase3, further linking dapagliflozin suppression of apoptosis and the AMPK/PKC/Nox signaling pathway (115). On the other hand, canagliflozin has been shown to accelerate the phosphorylation of AMPK (132). The phosphorylation of endothelial nitric oxide synthase (eNOS) and protein kinase B (Akt) (133), which could be related to apoptosis (132). As one of the causes of cardiovascular disease, eNOS can be observed in cardiomyocytes, where empagliflozin treatment triggers eNOS phosphorylation, confirming its cardioprotective abilities (104). The PI3K/Akt pathway is involved in cardiac remodeling, especially hypertrophic cardiomyopathy, *via* the mTOR pathway, and empagliflozin can suppress the cardiac hypertrophy caused by the PI3K/Akt/mTOR pathway (134). In other research, it is clear that empagliflozin can inhibit the PI3K/Akt/mTOR pathway by decreasing the phosphorylation of PI3K/Akt (135).

#### The relationship with connexin

Previous studies have demonstrated that dapagliflozin can delay the time of first VT, indicating that SGLT2 inhibitors exert antiarrhythmic effects mediated by Connexin 43 (Cx43) (136). Cx43 is a cardiomyocyte gap junction protein that facilitates cell communication by regulating the flow of electrical current (137). High phosphorylation of the Cx43 carboxyl terminal serine residue is essential for the residue of Cx43 (138). The mechanisms of Cx43 dephosphorylation, decreased Cx43 expression and Cx43 lateralization in the ventricle have not been defined. These processes have been reported to cause myocardial ischemia, arrhythmias and HF (139, 140). It has been found that cardiomyocyte death and electrical disorders are affected by Cx43 phosphorylation (141, 142). However, the mechanisms by which Cx43 phosphorylation triggers arrhythmias and the exact phosphorylation site(s) remain unclear (143). Lahnwong et al. showed that dapagliflozin significantly upregulated the phosphorylation of Cx43 and exerted anti-arrhythmia effect (136).

## Effects of SGLT2 inhibitors on other organs

The mechanisms by which SGLT2 inhibitors regulate kidney function are diverse. Previous studies have shown that the direct action of these inhibitors on renal vessels mediates their effects on renal function (144). Clinical studies have shown that empagliflozin can decrease the risk of malignant adverse renal outcomes such as acute renal failure (145). However, it has been linked to side effects such as genital infections and urinary infections (145). Experiments have shown that empagliflozin reduces the reabsorption of sodium, which in turn leads decreases ultrafiltration of the blood (146). Empagliflozin can also modulate the progression of kidney disease by altering arterial stiffness and uric acid levels (147). In past research, it was found that empagliflozin improved the indicators of arterial stiffness (147). However, its specific mechanism has not been clarified. Some scholars have suggested that the aforementioned effects may be related to the reduction of body weight and the control of glycaemia as well as the reduction of oxidative stress triggered by SGLT2 inhibitors (148). There is evidence that empagliflozin reduces the glomerular pressure and thus improves the glomerular filtration function in patients with type 1 diabetes (145) but does not prevent proteinuria (29). Recent studies have shown that SGLT2 inhibitors can affect glomerular filtration rate (GFR) by increasing the tone of glomerular afferent arterioles, thereby reducing glomerular plasma flow and intraglomerular pressure. The SGLT2 inhibitors can also reduce the GFR by decreasing the effective filtration pressure through the osmotic effect of non-reabsorbed glucose (149). The decline in GFR induced by SGLT2 inhibitors slows the progression of renal function decline similar to angiotensin (149). These suggests that SGLT2 inhibitors decrease GFR which is beneficial to the renal function. Furthermore, it has been suggested that the protective effects of kidney from SGLT2 inhibitors may be due to the decrease in intraglomerular pressure caused by osmotic diuresis.

Compared with other hypoglycemic drugs, SGLT2 inhibitors induce hypoglycemia through a mechanism independent of insulin regulation. SGLT2 inhibitors reduce body weight and increase fatty acid oxidation and ketone (150). Moreover, SGLT2 inhibitors contribute to the development of ketoacidosis by enhancing ketogenesis (29). Although SGLT2 inhibitors can regulate the generation of ketone bodies, the specific mechanism is unclear. Some clinical studies have shown that canagliflozin can ameliorate hepatic steatosis and non-alcoholic fatty liver disease (151). It activates lipolysis, increase fatty acid oxidation, and reduce hepatic steatosis by promoting the activation of FGF21 (152). On the other hand, it can directly activate the synthesis of hepatic ketone bodies by regulating glycogen consumption, thereby reducing the possibility of hepatic steatosis (152).

Atherosclerosis is a disease characterized by the accumulation of lipids, fibrous elements, and calcium deposits in the intima of arteries (153). Previous studies have shown that SGLT2 inhibitors can prevent the development of atherosclerosis and improve prognosis (154). In a previous study, SGLT2 inhibitors reduced atheromatous plaque size and arterial macrophage infiltration in diabetic mouse. Other studies have demonstrated the inhibitors can prevent atherosclerosis by reducing the formation of arterial foam cells (155). On the other hand, dapagliflozin can reduce the level of NLRP3 in arteries to protect the vasculature (156), but the specific mechanism has not been studied and still needs to be further explored. In recent studies, NLRP3 was found to be involved in the formation of inflammation in atherosclerosis, especially the formation of cholesterol crystals (157), which in turn activates caspase-1 to promote the production of IL-1β and IL-18. Together, these effects promote the development of inflammatory response (158). It is still not clear how SGLT2 inhibitors regulate the production of NLRP3. However, they have been reported to prevent cholesterol crystal formation in arteries, demonstrating their antiatherogenic effects (156).

## Future perspectives and conclusion

SGLT2 is mainly expressed in skeletal muscle and kidney, but not in heart (159). With the development of research, it has found that SGLT2 inhibitors can significantly reduce cardiovascular mortality (160, 161). SGLT1 is highly expressed in the heart (162). Therefore, SGLT2 inhibitors may regulate SGLT1 directly and attenuate the incidence of arrhythmia. However, the molecular mechanism by which SGLT2 inhibitors directly protect cardiac function remain unknown. Currently, studies have focused on investigating the indirect role of SGLT2 inhibitors in cardiovascular protection. In future, it will be interesting to determine whether SGLT2 inhibitors exert cardioprotective effects through SGLT1 receptors and the mechanism involved. In a recent study, SGLT2 inhibitors not only reduced the risk of HF hospitalization, but also reduced the odds of adverse event outcomes in HF (163).

In other reports, SGLT2 inhibitors significantly reduced the myocardial infarct size and reduced the risk of arrhythmia. Therefore, SGLT2 inhibitors are expected to play a key role in the protection against various cardiovascular diseases, especially HF, myocardial hypertrophy, myocardial infarction and arrhythmia. However, more studies are needed to elucidate its molecular mechanisms and targets of the cardioprotective effects of SGLT2 inhibitors, which may provide new therapeutic ideas for patients with cardiovascular risk factors. In addition, clarifying the cardiac protection mechanisms of SGLT2 inhibitors may be beneficial to bring cardiac protection into clinical life.

## Author contributions

JG contributed to literature researches. GZ, XW, and JL contributed to data collection. JG and GX were involved in the original draft of the manuscript. XY and YX participated in the review and editing of the manuscript. All authors have read and approved the final manuscript.
